# Zinc oxide resveratrol nanoparticles ameliorate testicular dysfunction due to levofloxacin-induced oxidative stress in rats

**DOI:** 10.1038/s41598-024-52830-w

**Published:** 2024-02-02

**Authors:** Naglaa F. Zaki, Sahar H. Orabi, Hend Mohamed Abdel-Bar, Hamed T. Elbaz, Reda M. S. Korany, Ayman K. Ismail, Walid M. Daoush, Maram H. Abduljabbar, Manal E. Alosaimi, Reem M. Alnemari, Heba H. Mahboub, Mohamed M. Ahmed

**Affiliations:** 1 Department of Biochemistry and Chemistry of Nutrition, Faculty of Veterinary Medicine, University of Sadat City, Sadat City, Egypt; 2 Department of Pharmaceutics, Faculty of Pharmacy, University of Sadat City, Sadat City, Egypt; 3 Department of Theriogenology, Faculty of Veterinary Medicine, University of Sadat City, Sadat City, Egypt; 4 Department of Pathology, Faculty of Veterinary Medicine, Cairo University, Giza, Egypt; 5 Department of Forensic Medicine and Toxicology, College of Veterinary Medicine, Suez Canal University, PO Box 41522, Ismailia, Egypt; 6 Department of Chemistry, College of Science, Imam Mohammad Ibn Saud Islamic University (IMSIU), P.O. Box 90950, 11623 Riyadh, Saudi Arabia; 7 Department of Production Technology, Faculty of Technology and Education, Helwan University, Saray-El Qoupa, El Sawah Street, Cairo, 11281 Egypt; 8 Department of Pharmacology and Toxicology, College of Pharmacy, Taif University, 21944 Taif, Saudi Arabia; 9 Department of Basic Sciences, College of Medicine, Princess Nourah bint Abdulrahman University, P.O. Box 84428, 11671 Riyadh, Saudi Arabia; 10 Department of Pharmaceutics and Pharmaceutical Technology, College of Pharmacy, Taif University, 21944 Taif, Saudi Arabia; 11 Department of Aquatic Animal Medicine, Faculty of Veterinary Medicine, Zagazig University, PO Box 44511, Zagazig, Sharkia Egypt

**Keywords:** Biochemistry, Microbiology, Biomarkers, Health care, Medical research, Molecular medicine, Pathogenesis, Nanoscience and technology

## Abstract

The present work is aimed to assess the protective influence of zinc oxide resveratrol nanoparticles against oxidative stress-associated testicular dysfunction. The number of 50 male albino rats were randomly separated into five groups (*n* = 10): Group I, control: rats gavage distilled water orally; Group II, Levofloxacin: rats that administered Levofloxacin (LFX) softened in distilled water at a dosage of 40 mg/kg^−1^ BW orally every other day; Group III, Zn-RSV: rats administered with Zn-RSV (zinc oxide resveratrol in distilled water at a dose 20 mg/kg^−1^ BW orally every other day; Group IV, (LFX + Zn-RSV): rats that were administered with Levofloxacin along with Zn-RSV nPs; Group V, Levofloxacin + Zn: rats were administered with Levofloxacin and Zno at a dose of 20 mg/kg^−1^ BW orally every other day as mentioned before. This study lasted for 2 months. Sera were collected to assess luteinizing hormone (LH), follicle-stimulating hormone (FSH), and testosterone values. Testicular tissues were utilized to evaluate levels of superoxide dismutase (SOD), nitric oxide (NO), malondialdehyde (MDA), and catalase (CAT). Semen samples were utilized to measure their quality (motility, concentration, and vitality). Histopathological and immune histochemical techniques investigated the morphological changes in the testis. Rats treated with Levofloxacin showed significantly lower levels of serum LH, testosterone, FSH, testicular enzymatic NO, catalase, SOD, BAX, and BCL-2 immune reactivity and sperm quality but significantly greater testicular malondialdehyde and caspase-3 immuno-reactivity Compared to both control and zinc oxide resveratrol treatment. Zinc oxide resveratrol nanoparticles ameliorated the harmful side effects of Levofloxacin. Improvements were more pronounced in the co-treatment (LFX + Zn-RSV) Zinc oxide resveratrol group than in the co-treatment (LFX + Zno) Zinc oxide group. Zinc oxide resveratrol nanoparticles could be a possible solution for levofloxacin oxidative stress-induced fertility problems.

## Introduction

A third-generation fluoroquinolone called levofloxacin (LFX), has moderate activity against aerobes and is active against the majority of aerobic Gram-positive and negative Bacterial strains. It prevents the DNA gyrase enzyme, which is necessary for DNA repair, recombination, replication, and transcription^1^. LFX has been used to treat community-acquired pneumonia, including strains of many bacteria that are multidrug-resistant, as well as complex and simple urinary tract infections^1^. Fluoroquinolones are a class of antibiotics frequently prescribed by doctors for the treatment of various bacterial infections when there is the presence of a significant number of leukocytes in the semen or before an IVF procedure, without taking into account any microbiological evidence of infection Al-Dujaily et al.^2^.

Quinolones are a class of antibacterial substances with a wide range of actions. Among other substances, quinolones, also known as fluoroquinolones, 4-quinolones, carboxylic acids or quinolone, include ciprofloxacin, levofloxacin, ofloxacin, perfloxacin, norfloxacin, fleroxacin, temofloxacin, enofloxacin and difloxacin^3^. Orally, these medications are readily absorbed^4^. It has been established that the active ingredient of the medicines can enter the seminal fluid and impact on the sperm cells directly, causing physiologic, metabolic, and/or genetic changes. Numerous medications have been reported to have an impact on sperm motility and count, which are frequently employed as measures of the quality of semen. Additionally, exposure to various xenobiotic substances can have potentially negative effects on male gametes and may result in male-mediated teratogenic consequences on the outcome of pregnancy^4^.

Nanoparticles (NPs) often referred to as particles with a size between 1 and 100 nm, which exhibit high surface area to volume ration. These unique physicochemical properties of the NPs have drawn a lot of attention as a result of their fast-expanding usage in nanomedicine, including the delivery of drugs or genes for the treatment lots of diseases^5^. The pros of NPs have been proven to be superior, increased efficacy against virus infections that are resistant to drugs, including increased potency at low concentrations, accessibility for surface modification and cost-effectiveness^6^.

In all physiological tissues, zinc (Zn) is an essential trace metal micronutrient due to its vital action during the proteins synthesis, nucleic acids besides neurogenesis. The Food and Drug Administration (FDA) has included zinc on its list of safe substances^6^. Zinc oxide nanoparticles (ZnO NPs), have been widely utilized in a variety of industrial industries, including alloys, ceramics, paints, and rubber, as well as in biological ones, such as medicine, cosmetics, sunscreens, and food additives. The vast range of uses for ZnO NPs has been linked to their distinctive physicochemical properties due to its range of the energy band gap including absorption of UV light which enhance its antibacterial action, Moreover, their catalytic, semi-conducting, and magnetic characteristics^7^.

In addition to being a cofactor for over 80 enzymes involved in protein synthesis and DNA transcription, zinc is a significant trace element. Given that Zn regulates numerous Zn-dependent enzymes including nuclear factor erythroid 2-associated factor 2, matrix metalloproteinases, and metallothionein, among many others, it is necessary for the testis and prostate to have high zinc concentrations in order to maintain their normal physiology. Zn is therefore essential for the growth and proliferation of germ cells^8^.

Testicular membranes and steroidogenic activity are altered by chronic stress, and oxidative damage is a major component in these modifications. The abundance of polyunsaturated fatty acids in testicular membranes makes them particularly sensitive to oxidative damage. Chronic stress causes changes in testicular membranes and testicular steroidogenic activity, and oxidative stress is a key factor contributing to these changes. Because testicular membranes are so abundant in polyunsaturated fatty acids, the testes are extremely vulnerable to oxidative stress^9^.

A polyphenolic substance called resveratrol [3,5,4′-trihydroxystilbene] naturally exists in grapes and plums. It comes in two different isomers, cis- and trans-resveratrol, and is either a monomer or an oligomer containing 2–4 monomer units. According to studies, resveratrol has antibacterial, anti-inflammatory, anti-oxidative, and antineoplastic actions. Additionally, it has antiatherogenic and antiangiogenic properties that allow it to contribute to the treatment of a number of cardiovascular illnesses. Additionally, it has been demonstrated that resveratrol has positive effects on both male and female rats' reproductive systems^10^ due to the fact that it can scavenge free radicals, resveratrol has also strong antioxidant effects that inhibit the production of ROS and prevent lipid peroxidation^11^. An antioxidant that occurs naturally in plants is called resveratrol. It is functions as an antioxidant primarily by scavenging free radicals or preventing their formation, preventing lipid peroxidation, and controlling the activity of enzymes that are involved in antioxidant synthesis (Xiong et al. 2021).

Resveratrol is extensively employed in the breeding industry as a feed supplement because of its biological antioxidative effect, which enhance meat quality and antioxidative capability by inhibiting lipid and protein oxidation. There is evidence that ingesting resveratrol significantly raised testicular antioxidant enzyme mRNA levels and superoxide dismutase (SOD) activity while reducing malondialdehyde (MDA) levels. The addition of resveratrol to the diet improved the meat quality of broilers by increasing catalase activity and antioxidative capacity while lowering MDA levels in the muscles^12^.

The objective of the current study was to demonstrate whether resveratrol-loaded ZnO- RSV NPs were effective in reducing the detrimental effects of oxidative stress caused by LFX on male fertility by examining oxidative damage parameters, gonadal hormone levels, semen quality indicators, and histological and immune-histochemical characteristics of testicular tissue.

## Material and methods

### Reagents

LFG, an Arabco Med firm, has donated LFX to us. (17,080,173). Sigma-Aldrich in the UK provided the Zinc nitrate hexahydrate, polyvinylpyrrolidone, sodium hydroxide, and nitric acid. The Chinese company Xian Sonwu Biotech Co., Ltd. provided the resveratrol. ELISA for diagnosis DiaSorin sold kits for measuring the values of luteinizing hormone, testosterone, and follicle stimulating hormone in serum. (Saluggia, Italy). A diagnostic kit was bought from Bio-diagnostic (Dokki, Giza, Egypt) for the evaluation of the antioxidant defense biomarkers. Such biomarkers include catalase (CAT. No: CA2517), superoxide dismutase (SOD) (SOD. No: SD2521), lipid peroxidation biomarker, MDA (MDA. No: MD.2529), and nitric oxide (NO. No: 2533).

### Preparation of resveratrol-loaded ZnO NPs

An approach called chemical precipitation was used to create ZnO NPs^13^. Zinc nitrate hexahydrate (500 mM) and polyinylpyrrolidone (PVP, 0.1% w/v) were dissolved in 100 mL of water, and sodium hydroxide (1 M) was gradually added while the mixture was continuously magnetically stirred at 1000 rpm for 4 h at 50 °C. (Abdel-Bar et al. 2021) The resulting dispersion underwent a 15-min centrifugation at 14,000 rpm. The recovered pellets were then progressively cleaned three times in ethanol and three times in deionized water. The fine powder that was obtained was then dried at 50 °C.

### Characterization of ZnO NPs

#### Diffraction analysis (X-ray) of ZnO NPs.

The synthesized ZnO NPs was investigated by XRD diffractometer of the model type (Netherlands-based Philips X'Pert) to analyze the crystal structure, crystallite size and other crystallographic parameters using X-ray powder diffractometer (XRD) technique. Cu K radiation of the 2 theta diffraction angle in the variety of 10–80° at 40 kV and 30 mA was used to conduct the analysis^14^. Using Scherrer's equation to estimate the crystallite size (d) as follows:

1 $${\text{d}} = \frac{K\lambda }{{\beta Cos\theta }}$$

where $\beta$= 0.15406 nm, is the line broadening at half the extreme intensity (FWHM) of the main peak in the XRD pattern, *K* is the dimensionless form factor of 0.9, and $\theta$ is Bragg's diffraction angle^14^.

#### FT-IR Spectroscopy

An FT-IR spectrometer was used to record the ZnO NPs' FT-IR spectrum. (JASCO 4000, USA). After blending 2 mg of the ZnO NPs with 100 mg KBr, the obtained mixture was pressed in a disc shape and the spectrum was recorded between 400 and 400 cm^−1^^15^.

#### Preparation and characterization of resveratrol-loaded ZnO NPs

ZnO -RSV NPs were synthesizes using surface adsorption technique, as previously described^16^. Stock solutions of ZnO NPs (10 mg/mL) and resveratrol (2 mg/mL) were mixed in acetone for 24 h at 1000 rpm in the dark using magnetic stirrer. The obtained combined solution was centrifuged three times at 14,000 rpm using deionized water to separate the nanoparticles. As noted earlier, dried ZnO-RSV NPs from the crop were used.

#### Determination of entrapment efficiency

By dissolving the synthesized ZnO -RSV NPs in appropriate volume of ethanol, it was possible to calculate the entrapment efficiency percentage (EE%) of resveratrol. By measuring the absorbance at 306 nm, the concentration of resveratrol was determined spectrophotometrically by using UV–vis double beam spectrophotometer of model Shimadzu 2450, Japan)^17^ and ZnO NPs as a blank. The entrapment efficiency percentage (EE%) can be estimated according to the following equation;

$${\text{EE}}{\mkern 1mu} \% = \frac{{Rsv\;content\;determined}}{{Total\;Rsv\;added}}*100$$

#### Determination of zeta potential

Deionized water was used to dilute the produced ZnO-RSV NPs (1:100), and then the Zeta potential was determined by using Nano-sizer ZS Series of model Malvern Instruments, UK) .

#### Transmission electron microscopy (TEM)

ZnO-RSV NPs suspended in deionized water was applied in a thin layer on a copper grid with a mesh size of 300, covered with carbon, and left to dry for ten minutes. As a result, filter paper was used to absorb any extra liquid. One drop of 1% phosphotungstic acid was used to stain the sample, which was then dried for five minutes and then investigated by using a TEM microscope of the model Jeol, JEM-1230, Japan.

#### Animals

Fifty albino Wistar male rats in good health condition, weighing between 105 and 110 g (6 to 8 weeks), were obtained from the Vac Sera Company (Dokki, Giza, Egypt). Rats were maintained in polypropylene cages with free entrance to water and a basal food (AL Wadi Company, ShibinAlqanater, AlQalyubia) under standard hygienic circumstances. Natural ventilation, a 12-h light/dark cycle, and an incubation temperature of 20–22 °C were all provided for the rats' housing. Prior to starting medication, rats had a 10-day acclimatization period.

#### Basal diet

Yellow corn, soy bean seeds and oil, monocalcium phosphate, limestone, sodium chloride, sodium bicarbonate, a combination of vitamins and minerals, and lecithin were all parts of the basic diet. 17% protein, 68.16% carbohydrates, 4.9% fat, 3.44% fiber, 3.5% salt mixture, 2% chloride, and 1% vitamin mixture made up the chemical makeup of the basic diet.

#### Experimental design

The experimental design and all procedures were performed following guidelines and principles and were permitted by the Research Ethics Committee of the Faculty of Veterinary Medicine, University of Sadat City, Egypt (VUSC-016–1-20). All experimental procedures were directed compliant with the Animal Research: Following the Vivo Experiments (ARRIVE) guidelines. Rats were randomly allocated into the following five groups of 10 rats: Group I, control: rats received gavage distilled water orally daily for 2 months; Group II, LFX: rats that administered LFX liquefied in distilled water at a dose of 40 mg/kg BW orally every other day for 2 months^18^; Group III, Zno-RSV: rats administered with (ZnO-RSV NPs in distilled water at a dose rate of 20 mg/kg BW orally every other day for 2 months^19^; Group IV, LFX + ZnO-RSV NPs: rats that were simultaneously administered with LFX along with ZnO-RSV NPs, as mentioned in the LFX and ZnO-RSV NPs treated groups; Group V, LFX + ZnO NPs : rats were administered with LFX its dose as mentioned before and ZnO at a concentration of 20 mg/kg BW orally daily for 2 months^19^.

#### Sampling

Animals were anesthetized at the end of the trial period (2 months). Rats were anesthetized by using phenobarbital at a dose of 45 mg/kg bw i.p. Capillary tubes were used to collect blood samples from the inner canthus of the eye. After allowing the blood samples to coagulate at room temperature, they underwent centrifugation for 15 min at 3,000 rpm. The hormonal assay was investigated using the clear supernatant serum, which was aspirated and kept at -20 °C. The two testicles of the rats were promptly removed once they were slaughtered. One testicle was used for histological analysis and immunohistochemistry and was stored in 10% neutral buffered formalin. For the purposes of measuring MDA, nitric oxide levels, catalase activity, and SOD activity, the other testicle was kept at—80 °C.

#### Serum hormone assays

Using a direct competitive immunoassay kit, serum testosterone was measured (Feldman et al. 2002). As previously indicated, quantitative detection of FSH and LH concentrations was carried out in accordance with the instructions for certain Sandwichchem luminescence immunoassay kits (Rabinovici et al. 1993). Values of serum hormones were calculated using commercial ELISA kits, and the assessment method based on the manufacturer's protocol. These included kits for assessment of LH (CAT. No. BC- 1,029, BioCheck, CA Inc.) and FSH (CAT. No. RH- 251, DSI, Italy Inc.). To measure the level of the testosterone hormone, the protocol depended on the competitive enzyme immunoassay procedure using a specific kit (CAT. No. CAN-TE-250, DBC, Canada Inc.). The level of testosterone in the samples was computed using the standard curve.

#### Testicular tissue antioxidant assays

One gram of tissue was used to create a homogenate in nine milliliters of PBS (1:10 W/V) in order to analyze the antioxidant defense system and lipid peroxidation biomarkers in testicular tissues. According to a prior description by Shawky et al.^20^, the tissue homogenate was created. In accordance with the manufacturer's recommendations, specific kits were utilized to measure the MDA concentration, catalase, nitric oxide, and SOD activity in tissue homogenate.

For bioassays, the supernatant was transferred to fresh tubes and stored at -80 °C^21^. SOD, nitric oxide, catalase, and MDA antioxidant/oxidant levels in testicular tissue were determined spectrophotometrically using specific universal kits (Bio-diagnostic Company, Cairo, Egypt) in accordance with the manufacturer's instructions.

#### Malondahyde (MDA)

Lipid peroxidation products, such as MDA, were determined in testicular homogenates using the colorimetric method following the technique designated by Ohkawa et al.^22^.

The MDA content was calculated as follows;

$${\text{MDA content }}\left( {{\text{nmol}}/{\text{g tissue}}} \right) \, = \, \left( {{\text{A sample}}/{\text{A standard}}} \right) \, \times \, \left( {{1}0/{\text{g tissue used}}} \right)$$

#### Superoxide dismutase

Colorimetric method was used for detecting SOD activity in testicular homogenates as previously described by Nishikimi et al.^23^ according to the following equation;

$${\text{SOD Activity }}\left( {{\text{U}}/{\text{g tissue}}} \right) \, = \, \% {\text{ inhibition }} \times { 3}.{75 } \times \, \left( {{\text{L}}/{\text{g tissue}}} \right).$$

#### Nitric oxide

The Biodiagnostic nitric oxide assay kit delivers an precise protocol for measuring nitrite level as an sign for nitric oxide release in biological fluids, which was determined according to the technique defined by Montgomery and Dymock^24^ as following;

$${\text{Nitrite in sample }}\left( {\mu {\text{mol}}/{\text{L}}} \right) \, = \, \left({{\text{A sample}}/{\text{A standard}}} \right) \, \times { 5}0$$

#### Catalase

Catalase activity was assessed in testicular homogenate depending on the method mentioned by Aebi^25^ as follows.

$${\text{Catalase activity }}\left( {{\text{U}}/{\text{g}}} \right) \, = \, \left({{\text{A standard }} - {\text{A sample}}/{\text{A standard}}} \right) \, \times {\text{ L}}/{\text{g tissue}}.$$

#### Epididymal sperm preparation

Epididymal sperm assessment was done using the conventional methods^26^. The dissected epididymal tail of rats were obtained and subjected to several cuts using sterile scissors and transferred to 1 mL of pre-warmed phosphate buffer saline (PBS). Gentle agitation was performed to the tearing tissue to make spermatozoa swim out into the pre-warmed PBS^27^. Each sample was incubated at 37 °C for 20 min for further sperm parameters analysis.

#### Evaluation of motility and sperm count

A 20-μL drop of the sample suspension was kept on a hygienic warm glass slide and enclosed with a pre-warmed cover slide at 37 °C. Many microscopical fields were inspected using phase-contrast microscope with hot stage (400 ×) magnification. Sperm motility (%) was expressed as proportion of progressive (rapid and slow) and non-progressive spermatozoa. While the total sperm count (10^6^/mL) in a drop of the resulting sperm suspension was done using a Neubauer hemocytometer and a light microscope as described by Yari et al.^28^

#### Evaluation of sperm viability

The viability was evaluated using eosin / nigrosine staining. In brief, the method was conducted by mixing sperm suspension; eosin and nigrosine stain in a ratio 1:2:3 respectively. Numerous films were arranged and at least 200 sperm cells were evaluated^27^.

Evaluation of live (unstained) and dead (pink stained) spermatozoa was done by using light microscopy under (400 ×) magnification  (Fig. I).

#### Histopathological examination

Testicular and epididymis samples were collected from the several experimental groups at the conclusion of the experiment, fixed in neutral buffered formalin 10%, cleaned, dehydrated, clarified, and then embedded in paraffin. For histological analysis, paraffin-embedded blocks were sectioned at a thickness of 5 m and stained with hematoxylin and eosin^29^. A light microscope was used to investigate the stained slices (Olympus BX50, Japan). Testis and epididymis histopathological alterations were noted and graded as no changes (0), mild (1), moderate (2), and severe (3) changes. Grading was based on the following percentages: 30% changes (mild change), 30%–50% (moderate change), and > 50% (severe change)^30^.

#### Immunohistochemical investigation

The indicated procedures were followed for performing an immunohistochemical study. After being deparaffinized in xylene, tissue pieces were rehydrated in various grades of alcohol. Pretreating the sections with a citrate buffer with a pH of 6 for 20 min enabled the antigen retrieval. Rabbit monoclonal anti-Bax antibody [E63] at a concentration of 1:250 (ab32503; Abcam, Cambridge, UK) and rabbit polyclonal anti-Bcl-2 antibody [ab59348; Abcam, Cambridge, UK] were incubated with sections for 2 h in a humid environment. 3,3′-diaminobenzidine tetrahydrochloride (DAB, Sigma) was utilized as a chromogen, and goat anti-rabbit IgG H&L (HRP) (ab205718; Abcam, Cambridge, UK) was utilized to incubate the sliced sections. The slides were then mounted with DPX and counterstained with hematoxylin. Slides for the negative controls were made by exchanging main antibodies by Orabi et al.^31^ and Baraka et al.^32^.

#### Evaluation of Bcl-2 and BAX proteins immunostaining

A total of five tissue slices from each group were used to assess the quantitative immunoreactivity of Bcl-2 and Bax. Under a high-power microscopic field (400), immunoreactivity was examined in 10 microscopical fields per section. The colour deconvolution picture J 1.52 p programme assessed the percentage of positively stained cells (%)(Wayne Rasband, National Institutes of Health, USA).

### Statistical analysis

One-way analysis of variance (ANOVA) was utilized to assess the significance of all data, which were then subjected to Duncan's post-hoc test for variance differences (P 0.05). Using SPSS, every statistical analysis was done. (SPSS version 13.0, IBM, Chicago, ILUSA).

### Ethics approval

The Faculty of Veterinary Medicine, University of Sadat City, Egypt's Research Ethics Committee authorized the experimental concept and all procedures (VUSC-016–1-20). All methods are reported in accordance with ARRIVE guidelines.

## Results

### Transmission electron microscope

**Figure 1:**
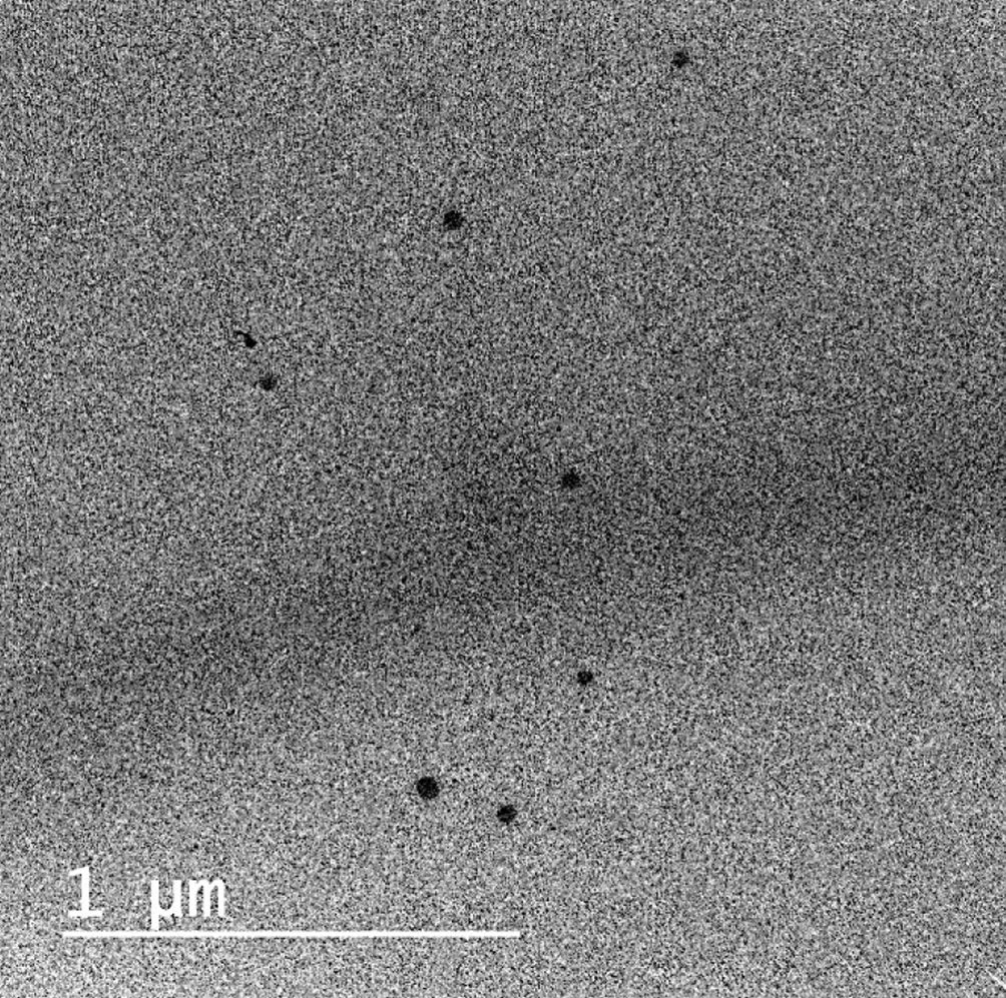
Transmission electron micrograph of RSV-Zno NPs. RSV-Zno nPs appeared as hexagonal nanostructures with crystallite size values consistent with XRD technique.

Morphological investigations of the synthesized ZnO-RSV NPs were visualized using TEM microscope. Figure 1 shows the TEM image of the ZnO-RSV NPs. It was observed from the results that the synthesized ZnO-RSV NPs have hexagonal particles shape of median particle size 20–30 nm which is matched with the estimated results obtained from the XRD analysis.

### Preparation and characterization of ZnO-RSV NPs

By virtue of its chemical structure, Resveratrol contains three hydroxyl groups that could possess an affinity for binding with ZnO NPs^33^. Table 1 shows that RSV EE% was 53.25% ± 5.98%. The slightly average value of EE% could be attributed to the RSV leakage during washing steps^16^. The measured value of the ZnO-RSV NPs Zeta potential was + 13.58 ± 2.66 mV. These findings are consistent with those of earlier investigations^34^. The positive charge of ZnO-RSV NPs could be due to the isoelectric point of ZnO NPs (pH 8.2) which is measurement in deionized water^35^.

**Table 1:** In vitro characterization of the prepared ZnO-RSV NPs.

Parameter	Crystallite size (nm)	Resveratrol EE %	Zeta potential (mV)
Result ± SEM	25.64 ± 1.5	53.25 ± 5.98	13.58 ± 2.66

X-ray diffraction analysis of ZnO NPs (Fig. 1) revealed that; the produced ZnO NPs pattern showed typically 2 theta peaks at 31.75°, 34.39°, 36.21°, 47.57°, 56.59°, 62.75°, 67.97°, and 69.02°. These peaks are confirming the presence of the crystal structure corresponding to the hexagonal wurtzite P63mc phase. These findings show that ZnO NPs were successfully synthesized^36^ and^37^. Also; the particle size of the synthesized ZnO NPs was 25.64 ± 1.5 nm as calculated by Scherrer’s equation (Table 1).

### FT-IR spectroscopy

**Figure 2:**
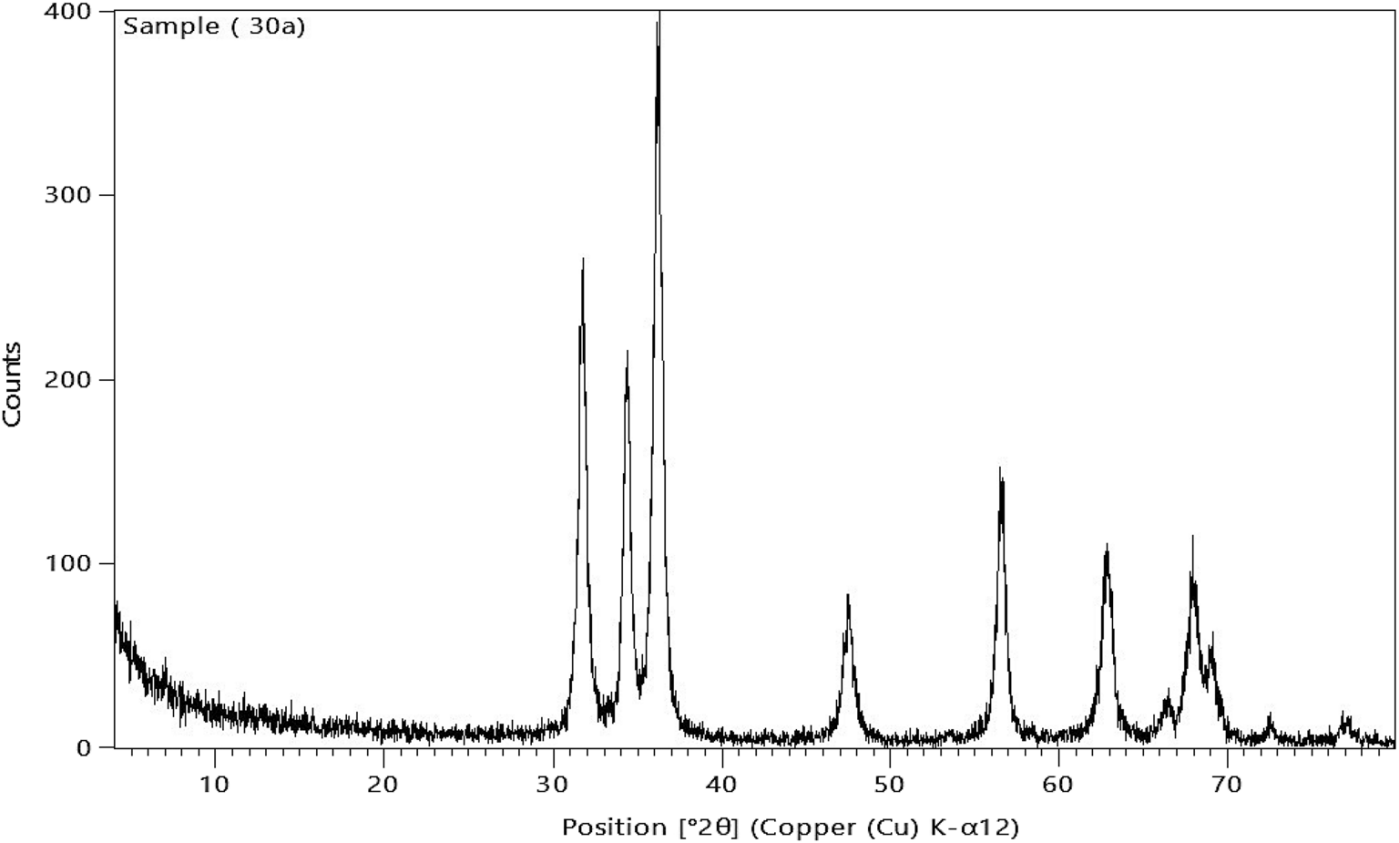
Characterization of the prepared Zno nPs with XRD. XRD pattern of the Zno nPs depicts the characteristic peaks of the hexagonal wurtzite P63mc crystal structure.

**Figure 3:**
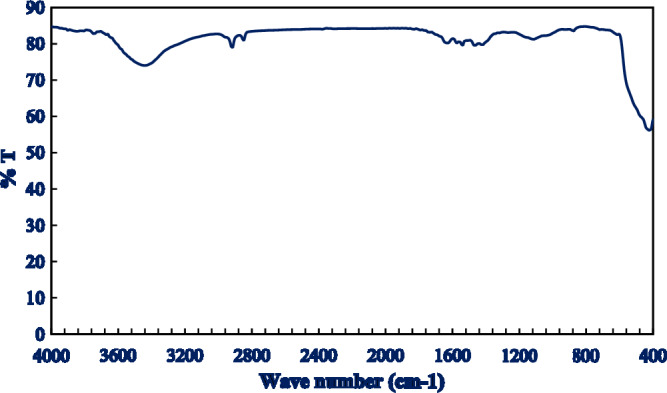
FT-IR Spectrum of the prepared Zno nPs. The FT-IR spectrum of the optimized Zno nPs revealed an obvious peak at 430 cm-1 assigned to Zn–O stretching.

Figures 2 and 3 showed that the characteristic bands observed at 3400 and 430 cm^−1^ can be attributed due to the O–H and Zn–O stretching, respectively, confirming the formation of ZnO NPs^38^. The presence of the OH broad band could be attributed due to presence of the intermolecular hydrogen bonding occurred with water by the possible moisture uptake from the atmosphere^39^. The indicated bands at 1650, 1430, and 1265 cm^−1^ are assigned to the stretching vibration of the C = O bond, the adjacent CH_2_ groups and the C-N in the pyrrole ring of PVP respectively^40^.

### Effects of LFX and ZnO-RSV NPs on serum hormone levels

Table 2 showed that oral administration of LFX in group II compared to the control group, caused a significant decline in FSH, LH, and testosterone. LFX + ZnO-RSV NPs and LFX + ZnO NPs had no significant effect compared to the LFX group. ZnO-RSV NPs had no effect on the serum testosterone, FSH, and LH concentration compared to the control group.

**Table 2:** Effects of LFX and ZnO-RSV NPs on serum hormone levels.

	Control	LFX treated	ZnO.RSV treated	LFX + ZnO-RSVtreated	LFX + ZnO treated
Testosterone (ng/ml)	6.89 ± 0.5a	3.77 ± 0.5b	7.7 ± 0.7a	4.22 ± 0.8b	4.3 ± 0.28b
LH (mLU/mL)	1.75 ± 0.12a	1.1 ± 0.06b	1.53 ± 0.17ab	1.49 ± 0.17ab	1.18 ± 0.03b
FSH (mLU/mL)	0.97 ± 0.18a	0.41 ± 0.08b	0.69 ± 0.08ab	0.57 ± 0.01ab	0.51 ± 0.11b

Values are expressed as means ± SEM, the mean difference is significant at *p* < 0.05. Data carrying different letters in the same row were significant FSH, follicular stimulating hormone, LH, Leutinizing hormone,

### Effects of LFX and ZnO-RSV NPs on antioxidant activities in testicular tissue

Table 3 showed that the administration of LFX in rats caused lipid peroxidation in testicular tissues, as indicated by the increased testicular MDA levels and decreased nitric oxide levels, which inhibit the antioxidant defense system of the testes as indicated by lower activities of testicular SOD and catalase enzymes compared to those of the control group. However, co-administration of ZnO-RSV NPs with LFX-treated rats reduced testicular MDA concentrations and increased testicular nitric oxide concentrations and activities of SOD and catalase in testicular tissues compared to those in the LFX-intoxicated group. However, rats Co-administered with LFX + ZnO NPs had significantly decreased MDA and increased SOD, catalase, and nitric oxide levels. Conversely, ZnO-RSV NPs had no significant effects on testicular concentrations of MDA and nitric oxide or activities of SOD, and catalase compared to the control group.

**Table 3:** Effects of LFX and ZnO-RSV NPs on antioxidant activities in testicular tissue.

	Control	LFX treated	ZnO-RSV treated	LFX + ZnO-RSV	LFX + ZnO-treated
MDA mmol/gtissue	2.69 ± 0.3d	15.44 ± 1.6a	2.64 ± 0.15d	6.52 ± 0.24c	9.78 ± 0.17b
SOD u/gtissue	169.6 ± 1.3a	54.8 ± 2.08d	168 ± 2.64a	124 ± 2.29b	111.4 ± 2.73c
CAT u/g.tissue	6.24 ± 0.5a	0.96 ± 0.07d	4.1 ± 0.22b	3.53 ± 0.23b	1.95 ± 0.08c
NO mmol/g.tissue	44.95 ± 0.4a	20.02 ± 0.6c	42.11 ± 0.7a	34.2 ± 0.7b	34.21b

Values are expressed as means ± SE, the mean difference is significant at *p* < 0.05 .data carrying different letters in the same row were significant difference SOD, Superoxide dismutase, NO, Nitric oxide, MDA, Malondialdehyde, CAT, catalase, LFX, Levofloxacin, ZnO, Zinc oxide nanoparticles, ZnO-RSV NPs.

### Effect of LFX and/or ZnO-RSV NPs on epididymal semen

The total sperm count, viability, and motility in the LFX-intoxicated rats were considerably lower than those in the control group. Co-administration LFX + ZnO-RSV NPs and LFX + ZnO NPs partially restored sperm viability while restoring the sperm cell count and motility to levels similar to those in the control group. The concentration, viability, and motility of sperm were unaffected by ZnO-RSV NPs treatment in mature male rats (Table 4).

**Table 4:** Effect of levofloxacin and/or ZnO-RSV NPs on epididymal semen picture.

	Control	LFX treated	ZnO-RSV treated	LFX + ZnO-RSV NPs	LFX + ZnO-treated
Count (× 10^6^)	52.5 ± 0.85^a^	28.17 ± 1.4^b^	63 ± 1.2^c^	53 ± 1.01^a^	60 ± 1.28^c^
Rapid motility (%) (Grade a)	21.67 ± 0.97^a^	11.67 ± 0.98^b^	35 ± 1.7^c^	19.16 ± 1.42^a^	23.33 ± 1.36^a^
Slow motility (%) (Grade b)	32.42 ± 1.98^a^	17.4 ± 1.03^b^	33.33 ± 1.54^a^	33.32 ± 1.95^a^	31.67 ± 3.1^a^
Non progressive motility (%) (Grade c)	30 ± 1.89^a^	27 ± 1.69^a^	21 ± 1.58^a^	30 ± 1.34^a^	30 ± 2.89^a^
Immotile sperm (%) (Grade d)	18 ± 1.69^a^	44 ± 2.31^b^	9 ± 1.58^c^	20 ± 1.33^a^	15 ± 2.04^a^
Total motility (%) (Grade a, b, c)	84.09 ± 4.85^a^	56 ± 3.7^b^	89.33 ± 4.82^c^	82.48 ± 4.71^a^	85 ± 7.35^a^
Viability (%)	81.67 ± 0.47^a^	54.33 ± 0.72^b^	91.33 ± 0.57^e^	82.33 ± 0.6^ac^	86.33 ± 0.43^d^

Values are expressed as means ± SE, the mean difference is significant at *p* < 0.05 .data carrying different letters in the same row were significant difference.

### Effects of LFX and ZnO-RSV NPs on histopathological findings

**Figure 4:**
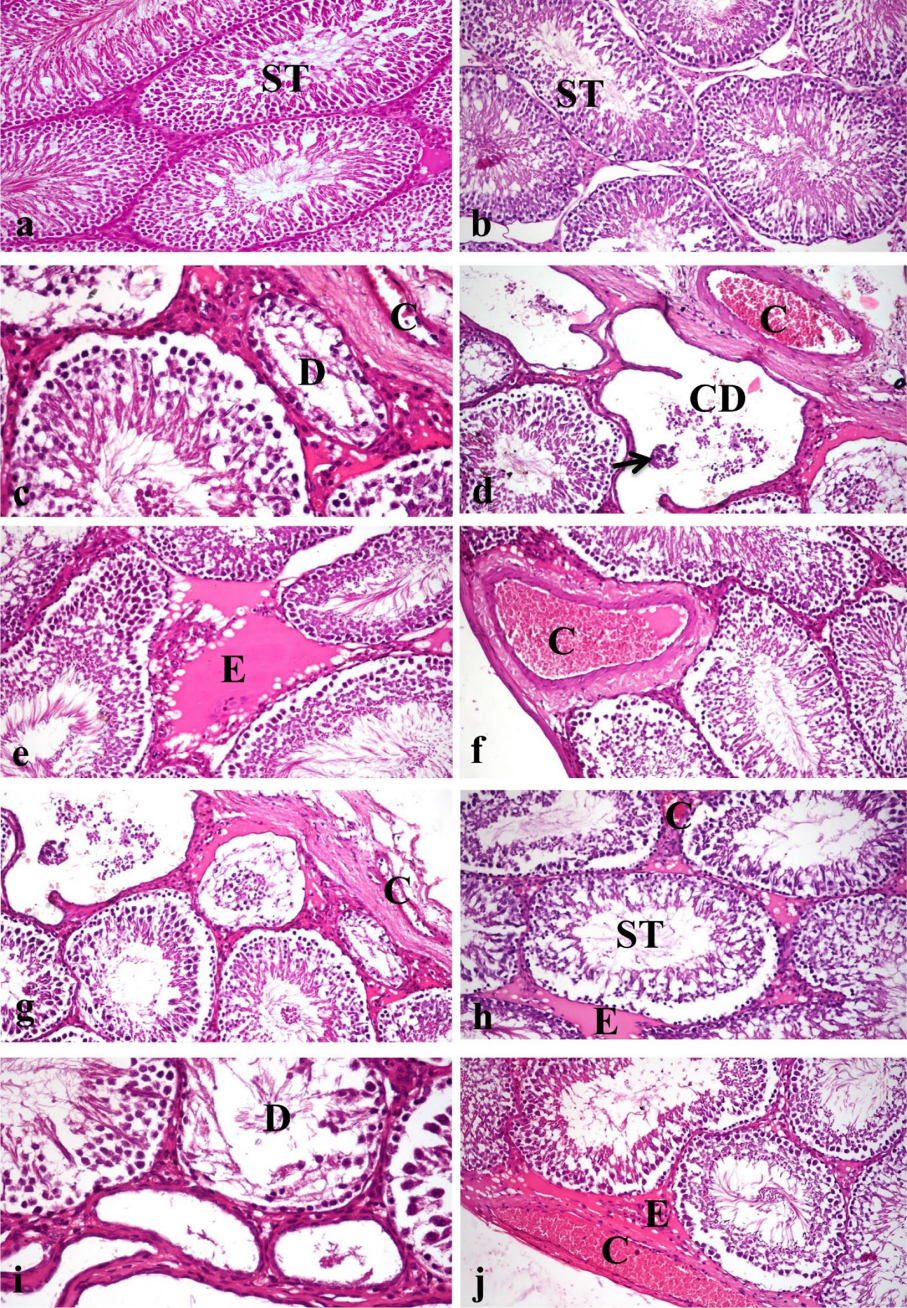
photomicrograph, testes of rat (**a**) Control group showing normal histological structure of seminiferous tubules (ST). (**b**) Group treated with zinc oxide resveratrol nanoparticle showing normal histological structure of seminiferous tubules (ST). (**c**) Group treated with levofloxacin showing degeneration of seminiferous tubules (D) and congestion of tunica albuginea blood vessels (C). (**d**) Group treated with levofloxacin showing cysticaly dilated seminiferous tubules which devoid of spermatogonial cells (CD), spermatid giant cell in tubular lumen (short arrow) with severe congested tunica albuginea blood vessels (C). (**e**) Group treated with levofloxacin, note edema of interstitial tissue (E). (**f**) Group treated with levofloxacin showing congestion of interstitial blood vessel (C). (**g**) Group treated with levofloxacin, note thickening of tunica albuginea with congestion of blood vessels (C). (**h**) Group treated with levofloxacin and co-administered with zinc oxide resveratrol nanoparticle, note normal histological structure of seminiferous tubules (ST), mild congestion of interstitial blood vessels (C) and mild interstitial edema (E). (**i**) Testis of group treated with levofloxacin and co-administered with zinc oxide nanoparticle showing testicular degeneration (D). (**j**) Testis of group treated with levofloxacin and co-administered with zinc oxide nanoparticle, note interstitial edema (E) and congestion of tunica albuginea blood vessel (C). (H&E X200).

Testes from the control group (Fig. 4a) and rats treated with ZnO-RSV NPs (Fig. 4b) showed the ordinary histological structure of their seminiferous tubules, interstitial tissue, and tunica albuginea. The LFX-intoxicated group showed testicular degeneration as indicated by the few spermatogenic cells lining the seminiferous tubules (Fig. 4c), the presence of spermatid giant cells with cystic dilatation of some seminiferous tubules, some seminiferous tubules showing an irregular outline of basement membrane as they were empty of any cellularity (Fig. 4d). Moreover, there was interstitial edema (Fig. 4e^),^ congestion in the blood vessels of the interstitial tissue (Fig. 4f), and thickening of the tunica albuginea with congestion of its blood vessels (Fig. 4g).

**Figure 5:**
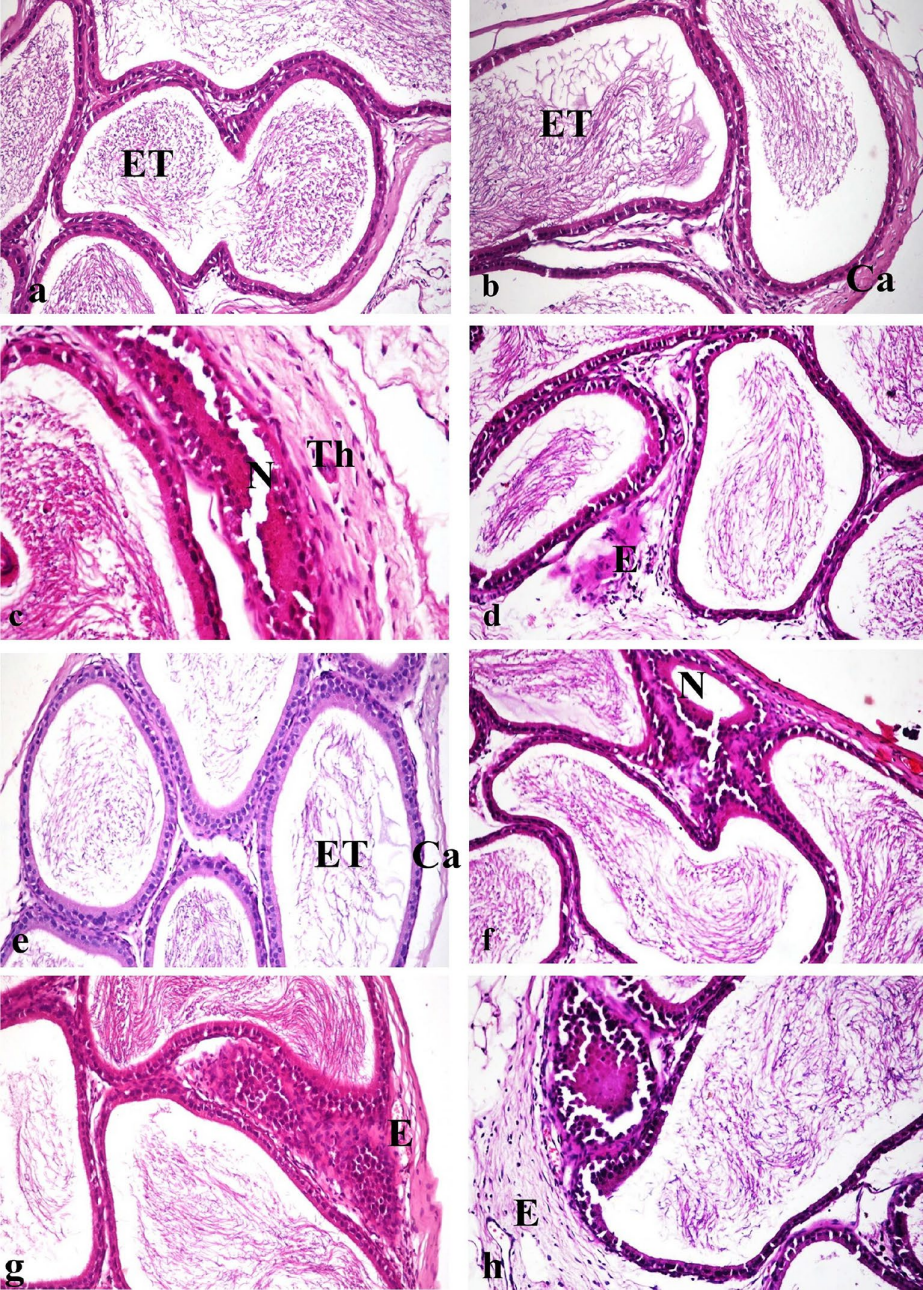
photomicrograph, epididymis of rat (**a**) Epididymis of control group showing normal histological structure of epididymal tubule with presence of sperms in the lumen (ET). (**b**) Epididymis of group treated with zinc oxide resveratrol nanoparticle showing normal histological structure of epididymal tubule (ET) and capsule (Ca). (**c**) Group treated with levofloxacin showing necrosis of tubular lining epithelium (N) with thickening of capsule (Th). (**d**) Group treated with levofloxacin showing edema of interstitial tissue with infiltration of mononuclear cells (E). (**e**) Group treated with levofloxacin and co-administered with zinc oxide resveratrol nanoparticle, note normal histological structure of epididymal tubule (ET) and capsule (Ca). (**f**) Group treated with levofloxacin and co-administered with zinc oxide nanoparticle, note necrosis of tubular lining epithelium (N) with congestion of capsular blood vessel (C). (**g**) Group treated with levofloxacin and co-administered with zinc oxide nanoparticle, note capsular edema and congestion (E). (**h**) Group treated with levofloxacin and co-administered with zinc oxide nanoparticle showing edema with mononuclear inflammatory cells infiltration in capsule (E) (H&E X200).

Rats Co-administered with LFX + ZnO-RSV NPs revealed a nearly normal histological structure of the testes with mild interstitial edema (Fig. 4h) and mild congestion of interstitial and tunica albuginea blood vessels. Rats Co-administered with LFX + ZnO-NPs showed mild improvement (Fig. 4i,j). Epididymis from the control group (Fig. 5a) and rats treated with ZnO-RSV NPs (Fig. 5b) showed normal histological structure. Rats with LFX intoxication showed necrosis of some epididymal tubules, which appeared devoid of sperm with capsule thickening (Fig. 5c), interstitial edema, and interstitial blood vessel congestion with infiltration of few mononuclear cells (Fig. 5d). Rats Co-administered with LFX + ZnO-RSV NPs showed the nearly standard histological structure of the epididymis (Fig. 5e). Those treated with LFX + ZnO NPs showed alterations similar to those in the LFX-intoxicated group (Fig. 5f, g, and h). The scoring of histopathological changes in the testes and epididymis of all treated groups is shown in Table 5.

**Table 5:** Scoring of histopathological alterations in testes and epididymis of all treated groups.

Lesion	Control	LFX treated	ZnO-RSV treated	LFX + ZnO-RSV	LFX + ZnO treated
-Testicular degeneration	0	2	0	0	2
-Edema of interstitial tissue	0	2	0	1	2
-Congestion of interstitial blood vessels	0	3	0	1	3
-Thickening and congestion of tunica albuginea	0	2	0	1	2
Necrosis of epididymal tubules	0	2	0	0	2
- Interstitial edema and congestion of epididymis	0	2	0	1	2
- Infiltration of mononuclear cells in interstitial tissue of epididymis	0	2	0	1	2

The score system was designed as: score 0 = absence of the lesion in all rats of the group (*n* = 5), score 1 = (< 30%), Score 2 = (< 30%–50%), score 3 = (> 50%).

### Effects of LFX and ZnO-RSV NPs on immunohistochemical findings

#### Immunohistochemical Bcl-2 and BAX proteins expression

**Figure 6:**
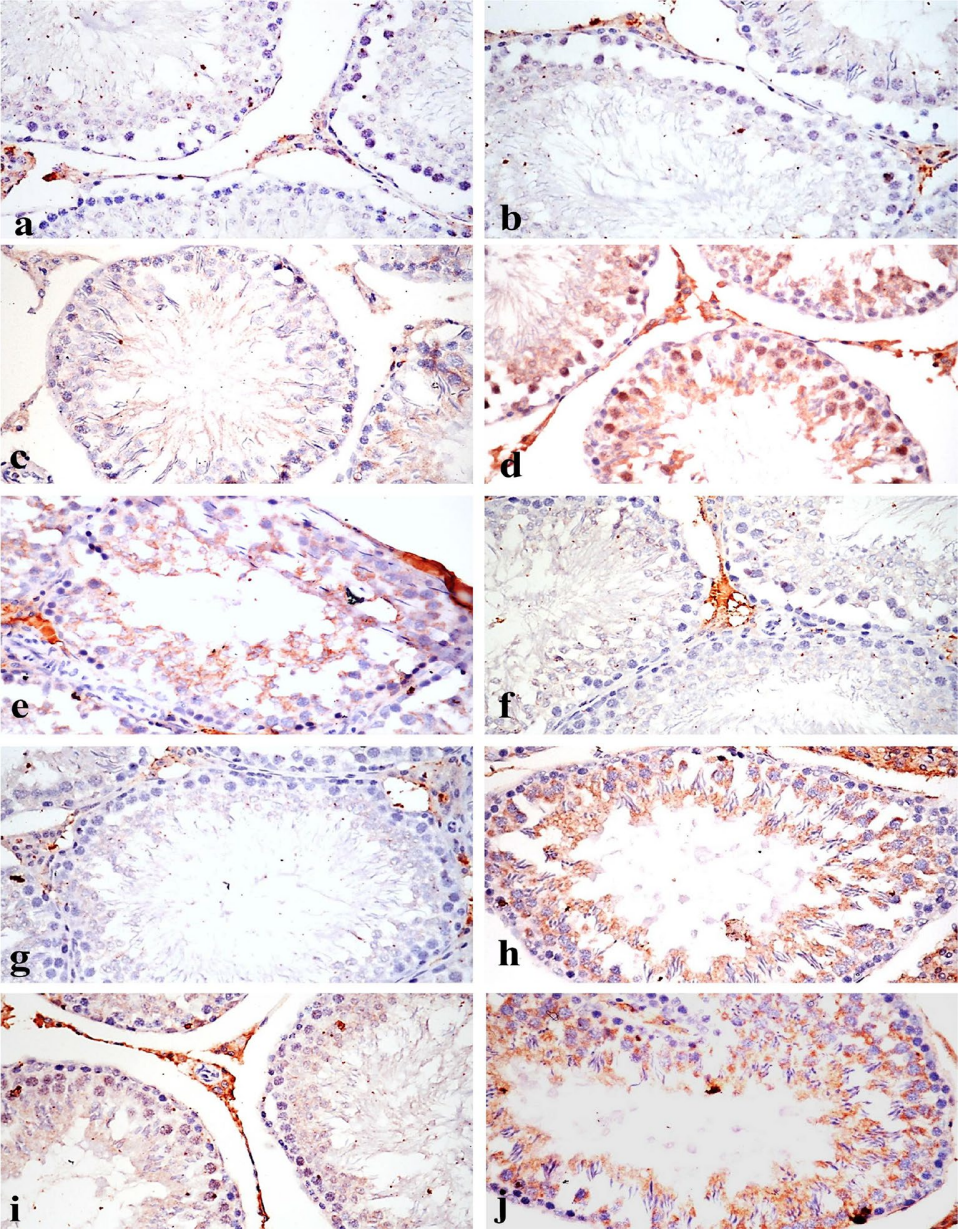
Immunostaining of BCL-2 and BAX in testes, (**a**) & (**b**) BCL-2 in control and zinc oxide resveratrol nanoparticle treated groups respectively showing no immune reactive cells (BCL-2 X200). (**c**) Levofloxacin treated groups showing weak positive expression (BCL-2 X200). (**d**) Group treated with levofloxacin and co-administered with zinc oxide resveratrol nanoparticle showing strong positive expression of BCL-2 (BCL-2 X200). (**e**) Group treated with levofloxacin and co-administered with zinc oxide nanoparticle showing weak positive expression of BCL-2 (BCL-2 X200). (**f**) & g) BAX in control and zinc oxide resveratrol nanoparticle treated groups respectively showing no immune reactive cells (BAX X200). (**h**) Levofloxacin treated groups showing strong positive expression of BAX (BAX X200). (**i**) Group treated with levofloxacin and co-administered with zinc oxide resveratrol nanoparticle showing weak positive immune expression of BAX (BAX X200). (**j**) Group treated with levofloxacin and co-administered with zinc oxide nanoparticle showing strong positive expression of BAX (BAX X200).

**Figure 7:**
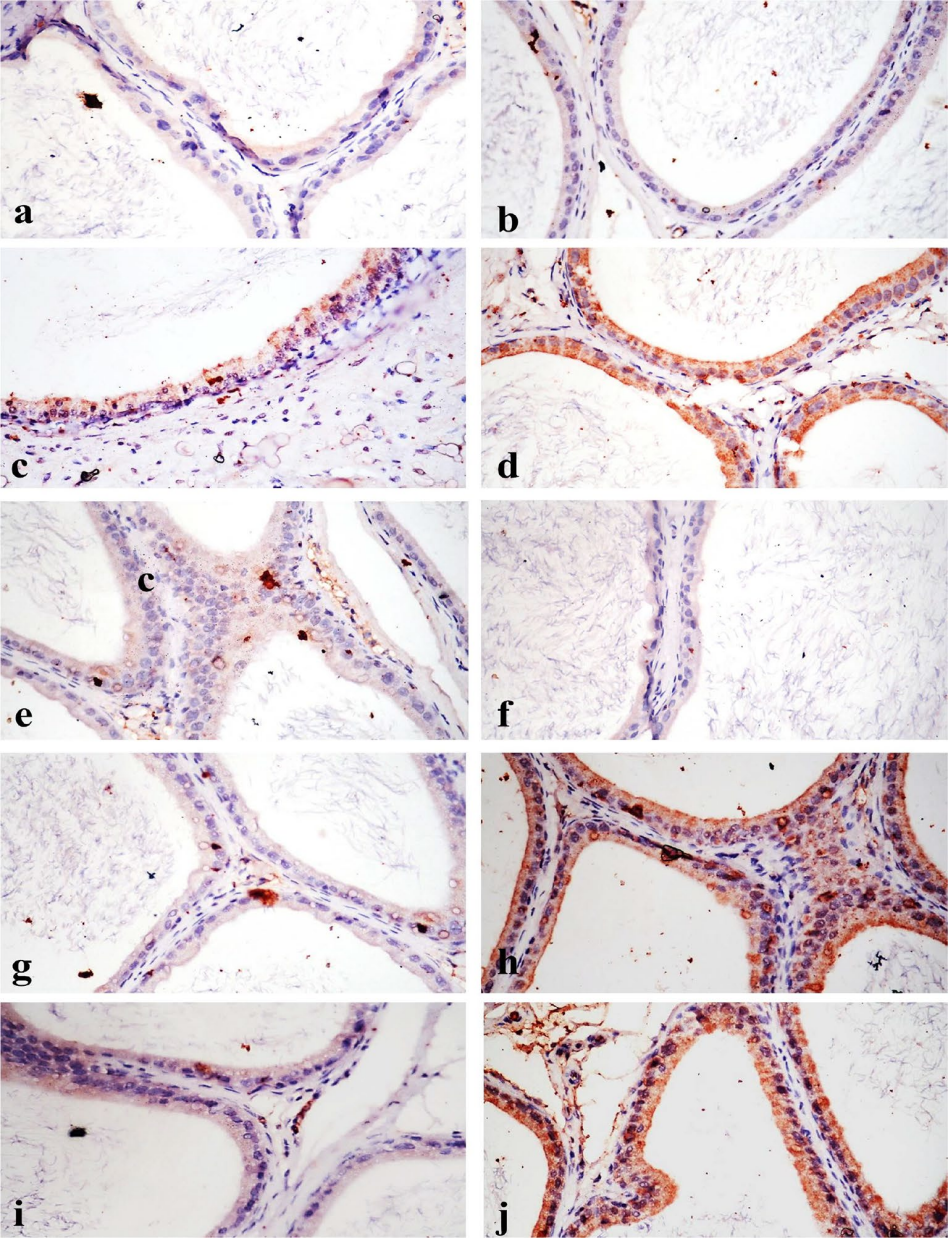
Immunostaining of BCL-2 and BAX in epididymis, (**a**) & (**b**) BCL-2 in control and zinc oxide resveratrol nanoparticle treated groups respectively showing no immune reactive cells (BCL-2 X200). (**c**) Levofloxacin treated groups showing weak positive expression (BCL-2 X200). (**d**) Group treated with levofloxacin and co-administered with zinc oxide resveratrol nanoparticle showing strong positive expression of BCL-2 (BCL-2 X200). (**e**) Group treated with levofloxacin and co-administered with zinc oxide nanoparticle showing weak positive expression of BCL-2 (BCL-2 X200). (**f**) & (**g**) BAX in control and zinc oxide resveratrol nanoparticle treated groups respectively showing no immune reactive cells (BAX X200). (**h**) Levofloxacin treated groups showing strong positive expression of BAX (BAX X200). (**i**) Group treated with levofloxacin and co-administered with zinc oxide resveratrol nanoparticle showing weak positive immune expression of BAX (BAX X200). (**j**) Group treated with levofloxacin and co-administered with zinc oxide nanoparticle showing strong positive expression of BAX (BAX X200).

Immunostaining expressions of BAX and Bcl-2 proteins in the testes and epididymis of various treated groups are illustrated in Tables 6 and 7, respectively. Immunostaining of Bcl-2 protein in the testes and epididymis showed negative effect (Figs. 6a and b, 7a and b) in both control and ZnO-RSV NPs treated groups, whereas BAX protein showed no immune reactive cells in the control and Zn –RSV treated groups (Figs. 6f and g, 7f and g) in all the organs under investigations. The group intoxicated with LFX showed weak immune effect of Bcl-2 protein (Figs. 6c and 7c) and strong expression of BAX protein (Figs. 6h^,^ and, 7h). Rats Co-administered with LFX + ZnO-RSV NPs showed strong effect of Bcl-2 (Figs. 6d and 7d) and weak effect of BAX proteins (Figs. 6i and 7i). Those Co-administered with LFX + ZnO-NPs showed immunostaining effect ranging from nil to a weak positive immune reaction of Bcl-2 (Figs. 6e and 7e) and strong expression of BAX protein (Figs. 6j, 7j).

**Table 6:** Area % of Bcl-2 and BAX expression in testes of different experimental groups.

	Control	LFX treated	ZnO-RSV treated	LFX + ZnO-RSV	LFX + ZnO treated
Bcl-2	0	0	19.2 ± 2.3^b^	53.2 ± 1.9^a^	23.6 ± 1.6^b^
BAX	0	0	48.7 ± 3.6^a^	21.3 ± 0.4^b^	51.9 ± 3.1^a^

Values are expressed as means ± SE, the mean difference is significant at *p* < 0.05 .data carrying different letters in the same row were significant difference.

**Table 7:** Area % of Bcl-2 and BAX expression in epididymis of different experimental ggroups.

	Control	LFX treated	ZnO-RSV treated	LFX + ZnO-RSV	LFX + ZnO treated
Bcl-2	0	0	14.4 ± 0.9^b^	49.1 ± 0.8^a^	13.7 ± 1.2^b^
BAX	0	0	51.2 ± 3.7^a^	17.2 ± 1.2^b^	47.9 ± 2.7^a^

Values are expressed as means ± SE, the mean difference is significant at *p* < 0.05 .data carrying different letters in the same row were significant difference.

## Discussion

Reactive oxygen species including hydrogen peroxide, superoxide anion, and hydroxyl radical have been observed to be produced by the antibiotic LFX. Through the processes of lipid peroxidation (LPO), protein modification, and DNA damage, these ROS induce oxidative stress and severe damage to macromolecules, tissues, and organs (Olayinka et al. 2014). Several processes that result in increased mitochondrial free radical generation, lipid peroxidation, and caspase activation are activated, which damages cells (Abdel- Alim et al. 2017). In current study, the result of the LFX intoxicated group revealed a significant decline in serum FSH, testosterone, LH and hormones that matched with the results of Ahmadi et al.^41^ that occurred due to increase lipid peroxidation in testes with evidence of increased level of MDA matched with Afalobi and Oyewo^18^, and that also agrees with our histopathological findings Concerning the levofloxacin-intoxicated group, revealed the presence of testicular degeneration in form of presence of spermatid giant cells with cystic dilatation of some seminiferous tubules, a few spermatogenic cells lining the seminiferous tubules, Interstitial edema and congested interstitial blood vessels were observed, certain seminiferous tubules lacked any cellularity, and tunica albuginea showed increased thickening and blood vessel congestion and this result was compatible with that recorded by Mokhimar et al.^42^. Previous research has reported that; some antibiotics result in the predicted death of cells by apoptosis, which can be crucial for decreasing the sperm motility. Rats given LFX generally developed quantitative sperm problems (lower sperm cell concentration) as well as qualitative sperm disorders (lower sperm viability and motility, as well as an increased proportion of morphological abnormalities)^2^. The use of Levofloxacin disturbs spermatogenesis and primary and secondary spermatocytes^41^. A promising mechanism of levofloxacin and ciprofloxacin-induced testicular cell injury is oxidative damage^42^. In the current investigation, LFX-treated rats shown a substantial drop in total sperm count, viability, and motility as compared with the control group, epididymis exhibited necrosis of some epididymal tubules and this agree with the results of Aral et al.^3^ and Al-Nazawi^43^ and Mokhimar et al.^42^ and epididymis's histopathology findings Levofloxacin intoxicated individuals displayed interstitial edema, congested interstitial blood vessels, and infiltration of a few mononuclear cells, as well as necrosis of certain epididymal tubules that seemed to be devoid of sperms was evident and this agrees with Mokhimar et al.^42^ and increases abnormal spermatozoa this agrees with Aral et al.^3^. Interstitial edema and congestion of interstitial blood vessels with infiltration of a few mononuclear cells were observed. In all previous studies on quinolone`s family as Ciprofloxacin, Norfloxacin, Ofloxacin and Levofloxacin, testicular toxicity was evident (Vahidi-eyrisofla et al. 2015). Levofloxacin caused testicular injury is due to oxidative stress and depletion of testicular antioxidant reserve (Olayinka et al. 2015). Group intoxicated with levofloxacin showed weak immune expression of Bcl-2 protein and strong expression of BAX protein. Apoptosis-mediated cell death brought by levofloxacin^41^. Bcl-2 has the unique role of extending cell survival by inhibiting apoptotic death. Bcl-2's special function is to prolong cell life by preventing apoptosis. Mammalian cells' equilibrium between suppressor and inducer gene products regulates apoptosis. According to recent research, the Bcl-2 protein interacts with proteins including Bax, Bcl-XL, Mcl-1, Bcl-XS, Bik, and Bad because their amino acid sequences are similar. Bax-mediated apoptosis appears to be prevented by Bcl-2 heterodimerization with Bax. Either apoptosis-suppressing proteins are activated or apoptosis-inducing proteins are blocked in order to sustain cell survival. Bax is mostly located in the cytoplasm, whereas Bcl-2 primarily localises the mitochondria, endoplasmic reticulum, and nuclear membrane. Bax travels from the cytosol to the membrane fractions in response to apoptotic stimuli, demonstrating that the redistribution of Bax is an early occurrence^44^.

Administration of ZnO-RSV NPs greatly mitigates the impact of LFX treatment on sperm parameters including viability, count and motility. These outcomes might be credited to antioxidant activity of the ZnO-RSV NPs as seen by a decline in MDA levels and an elevation in SOD, CAT, and NO activities in ZnO-RSV NPs treated rats. The antioxidant role of ZnO-RSV NPs may decline the disruptive, harmful impact of oxidative stress on hormone patterns.

Due to its radical scavenger activity, resveratrol has strong antioxidant effects that reduce the production of ROS and lipid peroxidation^11^. Rats, both male and female, both have favourable effects of RSV on their reproductive systems^10^. Adult male rats treated with ZnO-RSV NPs had no impact on the serum testosterone, FSH and LH concentration compared to the control group. Also, it did not affect serum reproductive hormones, sperm count, viability, motility and show normal histological architecture. Moreover, normal level of anti-oxidant enzymes as in the control group that agrees with^11^. This happens due to the biological anti-oxidative capabilities of ZnO-RSV NPs to improve semen quality and anti-oxidative capability by preventing lipid and protein oxidation. The levels of antioxidant enzymes and superoxide dismutase (SOD) activity in the testicles were dramatically increased by dietary resveratrol, and the amount of malondialdehyde (MDA) was lowered boosting the ability of semen to fight against free radicals, catalase activity, and malondialdehyde levels^12^. Moreover, ZnO-RSV NPs compared to the control group, had no appreciable effects on the testicular levels of MDA, NO, or the activities of SOD and catalase and this agrees with Singh et al.^45^. ZnO-RSV NPs showed the normal histological structure of interstitial tissue, tunica albuginea and seminiferous tubules. ZnO-RSV NPs demonstrated that the epididymis's histological structure was normal and that agrees with Fahim et al.^9^. Immunostaining of Bcl-2 protein in testes and epididymis the expression was negative in both control and ZnO-RSV NPs treated groups, while BAX protein showed no immune reactive cells in ZnO-RSV NPs treated groups and this agrees with Khodarahmian et al.^46^. Meanwhile, ZnO-RSV NPs nanoparticles are used in many studies as a strong antioxidant^47^. The group treated with levofloxacin and zinc oxide nanoparticles showed very mild or even no improvement in testicular toxicity. Also, (LFX + Zn) restored the sperm motility, sperm cell count, and sperm viability to levels comparable to the control group and co-treatment with (LFX + ZnO-RSV NPs) nanoparticles have no significant effect on serum reproductive hormones, antioxidant enzymes, and histological structure of testes comparable to the levofloxacin group. Co-administered levofloxacin with ZnO-RSV NPs showed strong expression of Bcl-2 and weak expression of BAX protein. Co-treatment with levofloxacin and ZnO-NPs showed immunostaining expression ranging from nil to weak positive immune-reaction of Bcl-2 and strong expression of BAX protein^41^. The outcomes of the current investigation revealed that the LFX group (GII) had significantly lower sperm motility, count, and viability as well as lower serum testosterone, LH, and FSH concentrations. This could be explained by greater oxidative stress in this group, as shown by the increase in MDA concentration and the decline in SOD, CAT, and NO activities, which are consistent with previously reported results (Al-Dujaily et al. 2018,Ahmadi et al. (2014) The histopathological abnormalities, weak immunological expression of the Bcl-2 protein, and robust expression of the BAX protein are all consistent with the changes in the LFX group's semen picture.

## Conclusion

The current perspective demonstrated that rats treated with ZnO-RSV NPs showed no noticeable differences compared to the control group. However, ZnO-RSV NPs ameliorated LFX-induced detrimental alterations in semen biomarkers, serum testosterone, FSH, LH, testicular antioxidants, histopathology, and immunoreactivity of protein markers. The administration of ZnO-RSV NPs for protection against LFX had better outcomes than its use as a form of treatment. The favorable impacts of ZnO-RSV NPs may result from its antioxidant activity, which counteracts LFX-induced oxidative stress, inhibits BCL-2 protein, and restores BAX protein immunoreactivity. ZnO-RSV NPs may be a suitable choice for dealing with the fertility problems associated with LFX.

### Supplementary Information

Supplementary Figure 1.

## Data Availability

All data generated or analyzed during this study are included in this published article and its supplementary information files.

## Abbreviations

FSH: Follicle-stimulating hormone
LFX: Levofloxacin
LH: Luteinizing hormone
MDA: Malondialdehyde
NPs: Nanoparticles
SOD: Superoxide dismutase
