# Supplementary material for: Zinc oxide resveratrol nanoparticles ameliorate testicular dysfunction due to levofloxacin-induced oxidative stress in rats
Source: Sci Rep. 2024 Feb 2;14:2752. doi: 10.1038/s41598-024-52830-w (PMC10837121; doi:10.1038/s41598-024-52830-w)
Supplement: Supplementary file 1 — Supplementary Figure 1. [file 41598_2024_52830_MOESM1_ESM.docx]

**Supplementary Figure 1**


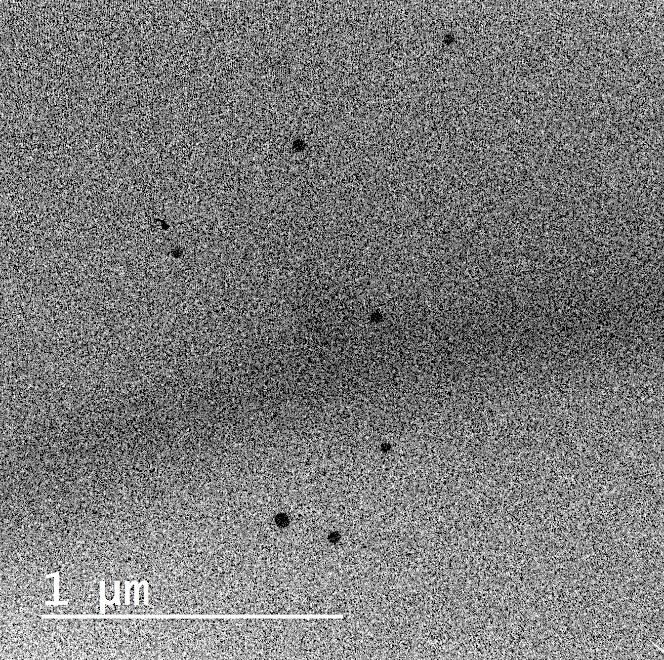


**Supplementary Figure**: Epididymal rat sperm stained with Eosin stain. (A) colourless live sperm (B) pink stained dead sperm.
